# Integrative web cloud computing and analytics using MiPair for design-based comparative analysis with paired microbiome data

**DOI:** 10.1038/s41598-022-25093-6

**Published:** 2022-11-28

**Authors:** Hyojung Jang, Hyunwook Koh, Won Gu, Byungkon Kang

**Affiliations:** 1 Department of Applied Mathematics and Statistics, The State University of New York, Korea, Incheon, South Korea; 2 Department of Computer Science, The State University of New York, Korea, Incheon, South Korea

**Keywords:** Computational biology and bioinformatics, Microbiology

## Abstract

Pairing (or blocking) is a design technique that is widely used in comparative microbiome studies to efficiently control for the effects of potential confounders (e.g., genetic, environmental, or behavioral factors). Some typical paired (block) designs for human microbiome studies are repeated measures designs that profile each subject’s microbiome twice (or more than twice) (1) for pre and post treatments to see the effects of a treatment on microbiome, or (2) for different organs of the body (e.g., gut, mouth, skin) to see the disparity in microbiome between (or across) body sites. Researchers have developed a sheer number of web-based tools for user-friendly microbiome data processing and analytics, though there is no web-based tool currently available for such paired microbiome studies. In this paper, we thus introduce an integrative web-based tool, named MiPair, for design-based comparative analysis with paired microbiome data. MiPair is a user-friendly web cloud service that is built with step-by-step data processing and analytic procedures for comparative analysis between (or across) groups or between baseline and other groups. MiPair employs parametric and non-parametric tests for complete or incomplete block designs to perform comparative analyses with respect to microbial ecology (alpha- and beta-diversity) and taxonomy (e.g., phylum, class, order, family, genus, species). We demonstrate its usage through an example clinical trial on the effects of antibiotics on gut microbiome. MiPair is an open-source software that can be run on our web server (http://mipair.micloud.kr) or on user’s computer (https://github.com/yj7599/mipairgit).

## Introduction

The human microbiome is the entire community of all microbes that inhabit different organs (e.g., gut, mouth, nose, skin, etc.) of the human body. The recent advance in next generation sequencing has enabled a faster, cheaper, and more precise quantification of the human microbiome. Then, the human microbiome field has rapidly emerged in both academia and industry. Researchers have found numerous significant discoveries on the effect of a treatment on the human microbiome^1–5^, the effect of an environmental/behavioral factor on the human microbiome^6,7^, and/or the effect of the human microbiome on human health or disease^3,8–14^. However, this would also indicate in contradiction that there can exist many potential confounders that lead to spurious discoveries.

One of the most efficient and practical ways to control for potential confounders is to use pairs (or blocks) at a design stage. Researchers can, for example, profile the human microbiome repeatedly per subject (1) before and after a treatment to see the effects of the treatment on microbiome^3,15–19^ or (2) for different organs of the body to see the disparity in microbiome between (or across) body sites^20–22^. Then, a study subject forms a pair/block for such repeatedly profiled microbiomes, in which potential confounders (e.g., genetic, environmental, or behavioral factors) are equally retained. Then, the use of appropriate statistical methods for such paired (block) designs can lead to valid and objective conclusions, not distorting the effects of a treatment on microbiome or the disparity in microbiome between (or across) body sites due to confounders.

Researchers have recently developed a sheer number of web-based data processing and analytic tools such as QIIME2^23^, PUMAA^24^, MicrobiomeAnalyst^25^, METAGENassist^26^, EzBioCloud^27^ and MiCloud^28^ for user-friendly microbiome data processing and analytics. These web-based tools have greatly accelerated the human microbiome studies with the facilities for cloud computing service and streamlined web environments that are easy-to-use for many people in a variety of disciplines (e.g., medicine, public health, biology, etc.). However, unfortunately, there is no web-based analytic tool currently available for paired microbiome studies. MiCloud^28^ is the web-based analytic tool that we developed for cross-sectional or longitudinal studies, yet even MiCloud^28^ can handle confounding effects only through covariate adjustments. Of course, covariate-adjusted analyses are important, though in practice, numerous potential confounders (e.g., genetic, environmental, or behavioral factors) can exist and they are usually lurking (i.e., nuisance variables that are unknown or not available in the data). Hence, it is often very hard to adjust them sufficiently in later statistical modeling.

**Figure 1:**
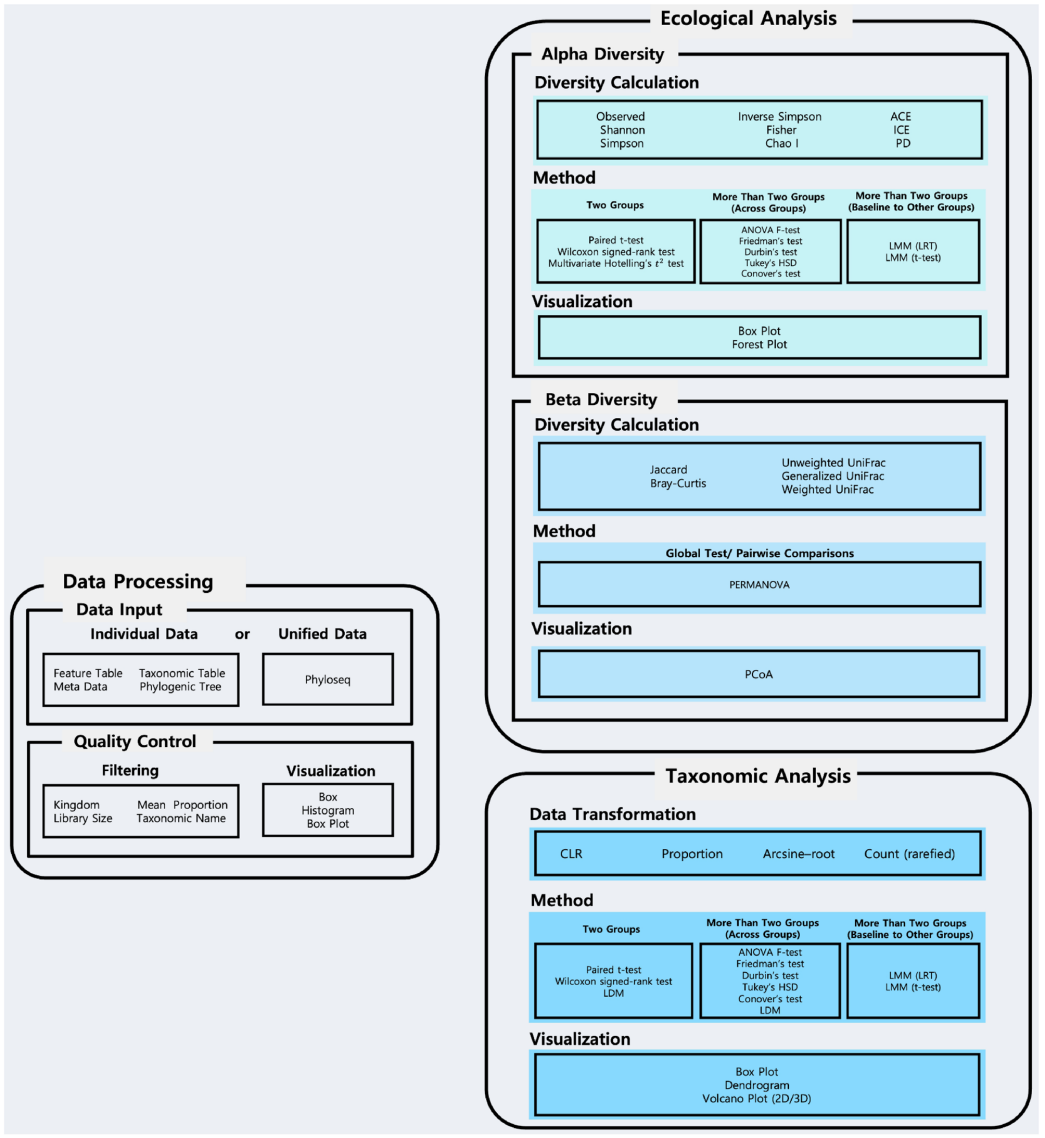
Overall workflow for MiPair. MiPair starts with a data processing component: data processing and then moves to two data analytic components: ecological analysis and taxonomic analysis.

Therefore, in this paper, we introduce an integrative web-based tool, named MiPair, for design-based comparative analysis with paired microbiome data. MiPair is a user-friendly web cloud service that enables comprehensive data processing and analysis sequentially for comparative analysis between (or across) groups or between baseline and other groups. MiPair employs parametric and non-parametric tests for complete (in which every block contains all possible levels of treatments or body sites) or incomplete (in which not every block contains all possible levels of treatments or body sites) block designs to perform comparative analyses with respect to microbial ecology (alpha- and beta-diversity) and taxonomy (e.g., phylum, class, order, family, genus, species) (Fig. 1). Thus, users can easily deal with comprehensive design-based data analyses with paired microbiome data. MiPair is an open-source software that can be run on our web server (http://mipair.micloud.kr) or alternatively on user’s computer (https://github.com/yj7599/mipairgit).

We organized the rest of the paper as follows. In “Results”, we delineate all individual data processing and analytic components (Fig. 1) with an example clinical trial on the effects of antibiotics on gut microbiome^3^. To brief, Zhang et al. collected fecal samples from non-obese diabetic mice and profiled their microbiomes using 16S rRNA amplicon sequencing^3^ and constructed microbiome data using QIIME^29^, whereas more details on this example study can be found in the original article^3^ The data were huge and motivated various study orientations, though for demonstration purposes, we reanalyzed a small portion of the data to see if the gut microbiome recovers from the time of a pulsed (macrolide) antibiotic administration (say, baseline) to 2 weeks and 4 weeks afterwards, respectively^3^ (see “Example”). In “Discussion”, we summarize the results, and importantly, discuss numerous potential applications of MiPair to other microbiome studies based on family/twin or matched designs. Finally, in “Materials and methods”, we described our web server, GitHub repository and the software packages that we used.

## Results

### Data processing: data input and quality control

We applied most parts of the Data Processing: Data Input and Quality Control component in MiCloud^28^ to MiPair. Yet, we additionally uploaded three new example datasets for a clinical trial on the effects of antibiotics on gut microbiome^3^ for users to easily catch up on. These three new example datasets are the ones for (1) a two-group comparison (a baseline group at the time of antibiotic administration and 2 weeks afterwards), (2) a three-group comparison (a baseline group at the time of antibiotic administration and 2 weeks and 4 weeks afterwards) based on a complete block design, where every subject contains all possible three levels of baseline, 2 weeks and 4 weeks afterwards, and (3) a three-group comparison (a baseline group at the time of antibiotic administration and 2 weeks and 4 weeks afterwards) based on an incomplete block design, where not every subject contains all possible three levels of baseline, 2 weeks and 4 weeks afterwards^3^. In the following sections, we describe the machinery of MiPair using the third example dataset for a three-group comparison based on an incomplete block design.

As in MiCloud^28^, users first need to upload four requisite data components: (1) feature table [i.e., count data for microbial features such as operational taxonomic units (OTUs) or amplicon sequence variants (ASVs)], (2) taxonomic table (i.e., taxonomic annotations on seven taxonomic ranks, kingdom/domain, phylum, class, order, family, genus, species), (3) metadata/sample information (e.g., treatment status, body sites, pair/block IDs) and (4) phylogenetic tree (i.e., rooted phylogenetic tree) using a unified phyloseq^30^ format or four individual files (Fig. 1).

Then, the data go through quality controls with respect to (1) a kingdom of interest [‘Bacteria’ (default) for 16S data, ‘Fungi’ for ITS data, or any other kingdom of interest for shotgun metagenomic data], (2) a library size for the samples to be removed [i.e., the samples that have a library size/total read count lower than 2000 (default) are removed], (3) a mean proportion for the features (OTUs or ASVs) to be removed [i.e., the microbial features that have a mean proportion lower than 0.002% (default) are removed] and (4) erroneous taxonomic names to be removed (Fig. 1).

MiPair displays summary data [sample size, numbers of features (OTUs, ASVs), phyla, classes, orders, families, genera, and species] using boxes, and data distributions using interactive histograms and box plots before and after quality controls.

#### Example

**Figure 2:**
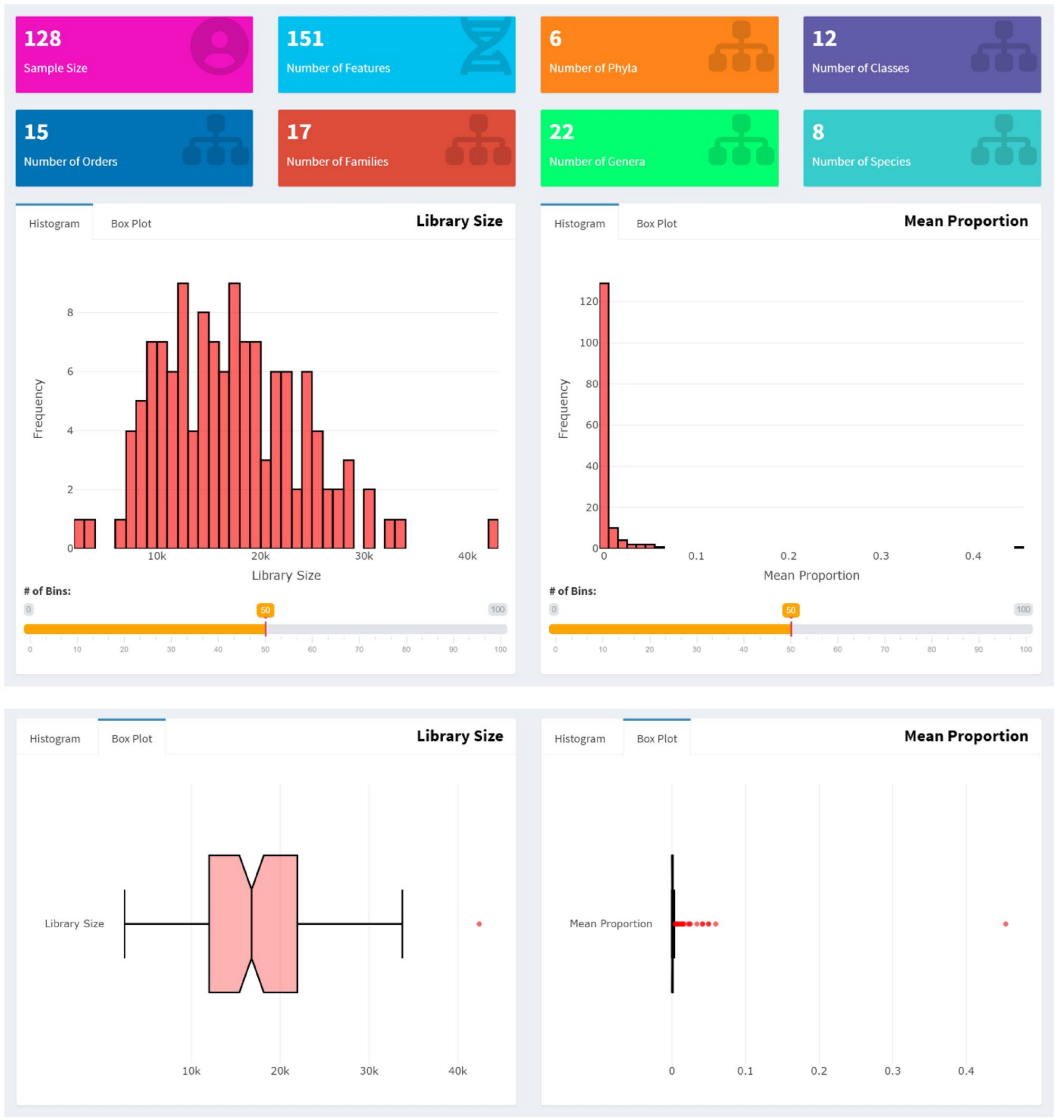
The results after the quality controls of MiPair. MiPair displays summary data (sample size, numbers of features (OTUs, ASVs), phyla, classes, orders, families, genera, and species) using boxes and visualizes the distributions of library sizes across samples and mean proportions across microbial features using histograms and box plots.

We uploaded the data for a three-group comparison based on an incomplete block design and applied the default quality control settings. Then, we rescued 151 features, 6 phyla, 12 classes, 15 orders, 17 families, 22 genera and 8 species for 128 samples (Fig. 2).

### Ecological analysis: diversity calculation

As in MiCloud^28^, MiPair considers a breadth of alpha- and beta-diversity indices that properly modulate the richness and evenness in diversity while reflecting phylogenetic tree information or not^31–34^. The alpha-diversity indices that MiPair calculates are Observed, Shannon^35^, Simpson^36^, Inverse Simpson^36^, Fisher^37^, Chao1^38^, abundance-based coverage estimator (ACE)^39^, incidence-based coverage estimator (ICE)^40^ and phylogenetic diversity (PD)^41^ indices. The beta-diversity indices that MiPair calculates are Jaccard dissimilarity^42^, Bray–Curtis dissimilarity^43^, Unweighted UniFrac distance^44^, Generalized UniFrac distance^45^ and Weighted UniFrac distance^46^ (Fig. 1) indices. Users can download those alpha- and beta-diversity indices for reference.

### Ecological analysis: alpha diversity

MiPair performs comparative analysis in alpha-diversity between (or across) groups (i.e., pre-treatment and post-treatment group(s), different body sites). Users first need to choose a primary variable of interest (i.e., a factor variable that contains multiple groups/levels of treatments or body sites). Then, MiPair lists groups/levels in a chosen primary variable and ask to choose at least two groups/levels to be compared. Then, users need to choose a variable for pair/block IDs (e.g., subjects IDs for pre and post treatments or body sites). Then, MiPair compares two groups or more than two groups (across groups or a baseline group to each of the other groups) in alpha-diversity (Fig. 1) as follows.

#### Two-group comparison

The parametric Paired t-test or the non-parametric Wilcoxon signed-rank test (default)^47^ can be employed to see if two groups have the same distribution for each alpha-diversity index (${H}_{0}$) or if they have different distributions (${H}_{1}$). For omnibus testing to see if the two groups have the same distribution across all alpha-diversity indices (${H}_{0}$) or if they have different distributions for at least one alpha-diversity index (${H}_{1}$), the multivariate Hotelling’s t-squared test^48^ can also be employed. MiPair visualizes the results using box plots and/or forest plots.

#### More than two-group comparison (across groups)

For the parametric inference, the repeated measures analysis of variance (ANOVA) F-test for global testing (to see if all groups have the same distribution for each alpha-diversity index (${H}_{0}$) or if at least one group has a different distribution (${H}_{1}$)) with the Tukey’s honestly significant difference (HSD) test^49^ for post-hoc comparisons (to test all possible pairs of groups, individually) can be employed. For the non-parametric inference in complete block designs, the Friedman’s test^50^ for global testing with the Conover’s test^51^ for post-hoc comparisons can be employed. For the non-parametric inference in incomplete block designs, the Durbin’s test for global testing with the Conover’s test^51^ for post-hoc comparisons can be employed. MiPair visualizes the results using box plots.

#### More than two-group comparison (baseline to other groups)

The likelihood ratio test (LRT) for global testing with the t-test for pairwise comparisons from a baseline group to each of the other groups based on the parametric linear mixed model (LMM)^52^ can be employed. MiPair visualizes the results using box plots.

#### Example

**Figure 3:**
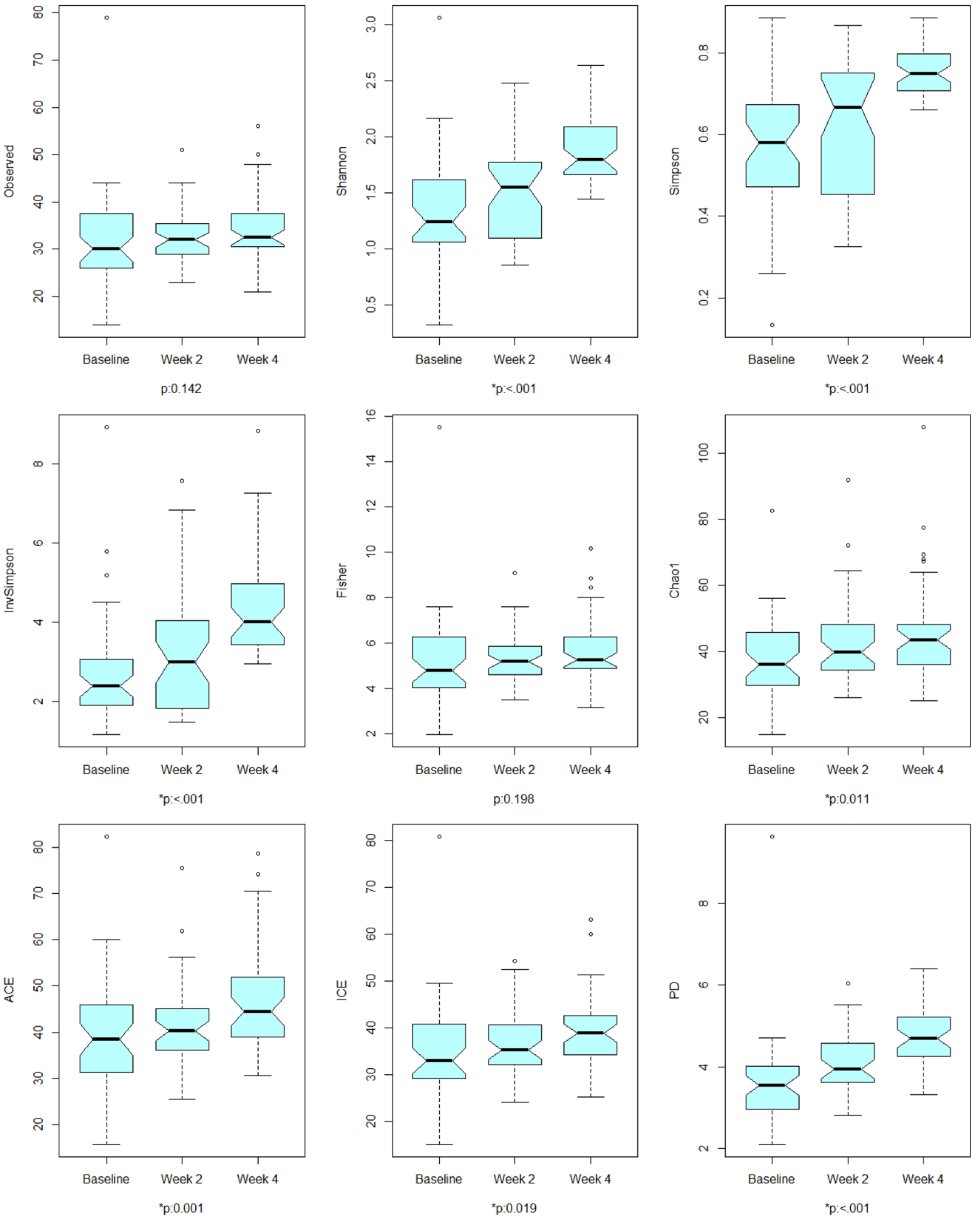
The results for comparitive analysis in alpha-diversity (global test). The p-values were calculated using LRT based on LMM for global testing to see if all groups have the same distribution in each alpha-diversity index (${H}_{0}$) or if at least one group has a different distribution in each alpha-diversity index (${H}_{0}$). *p represents statistical significance at the level of 5%.

We performed comparative analysis in alpha-diversity from the baseline group at the time of antibiotic administration to 2 weeks and 4 weeks afterwards^3^ using LMM for global testing (Fig. 3) and pairwise comparisons (Table 1). We found significant disparity in alpha-diversity for at least one group across the three groups with respect to Shannon, Simpson, Inverse Simpson, Chao 1, ACE, ICE and PD at the significance level of 5% (Fig. 3). We further observed that the alpha-diversity was significantly enriched 2 weeks afterwards with respect to Shannon and PD and 4 weeks afterwards with respect to Shannon, Simpson, Inverse Simpson, Chao 1, ACE, ICE and PD at the significance level of 5% (Table 1).

**Table 1:** The results for comparitive analysis in alpha-diversity (pairwise comparisons). *Ref represents the reference/baseline group, Com represents the comparison group, Est and SE represent the estimated regression coefficient and its standard error, t represents the t statistic value, and Adj. P-value represents the FDR adjusted P-value.

Alpha-diversity index	Ref	Com	Est	SE	t	Adj. P-value
Observed	Baseline	Week 2	1.568	1.164	0.972	0.334
	Baseline	Week 4	3.229	1.636	1.973	0.103
Shannon	Baseline	Week 2	0.167	0.076	2.195	0.031
	Baseline	Week 4	0.545	0.077	7.086	< 0.001
Simpson	Baseline	Week 2	0.051	0.027	1.888	0.063
	Baseline	Week 4	0.190	0.027	6.964	< 0.001
InvSimpson	Baseline	Week 2	0.462	0.264	1.745	0.085
	Baseline	Week 4	1.650	0.268	6.148	< 0.001
Fisher	Baseline	Week 2	0.249	0.327	0.764	0.447
	Baseline	Week 4	0.592	0.331	1.787	0.155
Chao1	Baseline	Week 2	4.882	2.874	1.699	0.092
	Baseline	Week 4	8.778	2.909	3.017	0.006
ACE	Baseline	Week 2	3.197	2.396	1.334	0.186
	Baseline	Week 4	8.935	2.426	3.683	0.001
ICE	Baseline	Week 2	2.091	1.738	1.203	0.232
	Baseline	Week 4	5.004	1.762	2.840	0.011
PD	Baseline	Week 2	0.484	0.163	2.967	0.004
	Baseline	Week 4	1.098	0.166	6.629	< 0.001

### Ecological analysis: beta diversity

MiPair performs comparative analysis in beta-diversity between (or across) groups (i.e., pre-treatment and post-treatment group(s), different body sites). As in Alpha Diversity, users first need to choose a primary variable of interest (i.e., a factor variable that contains multiple groups/levels of treatments or body sites). Then, MiPair lists groups/levels in a chosen primary variable and ask to choose at least two groups/levels to be compared. Then, users need to choose a variable for pair/block IDs (e.g., subjects IDs for pre and post treatments or body sites). Then, MiPair compares two groups or more than two groups (across groups or a baseline group to each of the other groups) in beta-diversity (Fig. 1) as follows.

#### Two-group comparison

The nonparametric permutational multivariate analysis of variance (PERMANOVA)^53,54^ for paired microbiome designs can be employed to see if two groups have the same microbiome composition for each beta-diversity index (${H}_{0}$) or if they have different microbiome compositions (${H}_{1}$). MiPair visualizes the results using principal coordinate analysis (PCoA) plots^55^.

#### More than two-group comparison (across groups)

MiPair employs PERMANOVA^53,54^ for global testing to see if all groups have the same microbiome composition for each beta-diversity index (${H}_{0}$) or if at least one group has a different microbiome composition (${H}_{1}$), and also for pairwise comparisons for all possible pairs of groups individually applying the Benjamini–Hochberg (BH) procedures^56^ to control for false discovery rate (FDR). MiPair visualizes the results using PCoA plots^55^.

#### More than two-group comparison (baseline to other groups)

MiPair employs PERMANOVA^53,54^ for global testing, and also for pairwise comparisons for all possible pairs of a baseline and each of the other groups individually applying the BH procedures^56^ to control for FDR. MiPair visualizes the results using PCoA plots^55^.

#### Example

**Figure 4:**
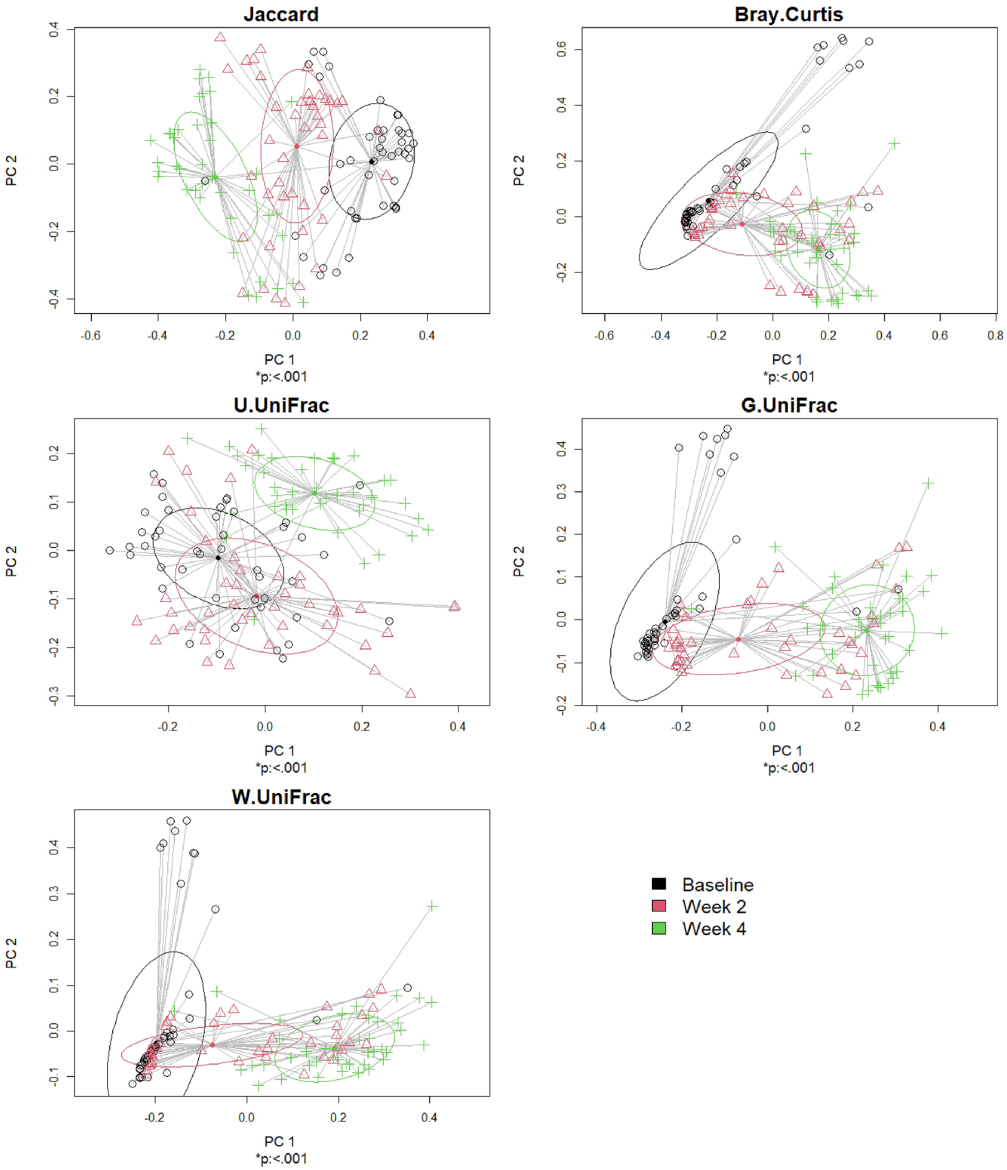
The results for comparitive analysis in beta-diversity (global test). The p-values were calculated using PERMANOVA for global testing if all groups have the same microbiome composition in each beta-diversity index (${H}_{0}$) or if at least one group has a different microbiome composition in each beta-diversity index (${H}_{1}$). *p represents statistical significance at the level of 5%.

We performed comparative analysis in beta-diversity from the baseline group at the time of antibiotic administration to 2 weeks and 4 weeks afterwards^3^. We found significant disparity in beta-diversity for at least one group across the three groups with respect to all the surveyed beta-diversity indices at the significance level of 5% (Fig. 4). We further observed significant disparity in beta-diversity for all possible pairs of the baseline group and each of the other two groups (2 weeks and 4 weeks afterwards) with respect to all the surveyed beta-diversity indices at the significance level of 5% (Table 2).

**Table 2:** The results for comparitive analysis in beta-diversity (pairwise comparisons). *Ref represents the reference/baseline group, Com represents the comparison group, F represents the F statistic value, and Adj. P-value represents the FDR adjusted P-value.

Beta-diversity index	Ref	Com	F	Adj. P-value
Jaccard	Baseline	Week 2	11.828	< 0.001
	Baseline	Week 4	19.136	< 0.001
Bray.Curtis	Baseline	Week 2	9.468	< 0.001
	Baseline	Week 4	21.565	< 0.001
U.UniFrac	Baseline	Week 2	8.226	< 0.001
	Baseline	Week 4	14.584	< 0.001
G.UniFrac	Baseline	Week 2	15.690	< 0.001
	Baseline	Week 4	44.064	< 0.001
W.UniFrac	Baseline	Week 2	12.951	< 0.001
	Baseline	Week 4	53.649	< 0.001

### Taxonomic analysis: data transformation

For taxonomic analyses at each of the seven taxonomic ranks (phylum, class, order, family, genus and species), MiPair first transforms the original count data into four different data forms, (1) centered log ratio (CLR)^57^ to normalize the data and relax the compositional constraint, (2) proportion to control for varying library sizes across samples, (3) arcsine-root to control for varying library sizes across samples and stabilize the variability across samples (4) count (rarefied) ^58^ to control for varying library sizes across samples and use counts as the data form. These data forms have all been widely used, and each of them has both advantages and disadvantages. Hence, it is hard to conclude which data form is superior to the other data forms in all contexts. We set up all such data forms as user options with no default setting. Users can download the original and transformed datasets for reference.

### Taxonomic analysis: differential abundance analysis

MiPair performs comparative analysis in each microbial taxon at each of the seven taxonomic ranks (phylum, class, order, family, genus and species). Users first need to choose a data format among CLR ^57^, proportion, arcsine-root and count (rarefied)^58^ (Fig. 1). Then, as in Alpha Diversity and Beta Diversity, users need to choose a primary variable of interest (i.e., a factor variable that contains multiple groups/levels of treatments or body sites). Then, MiPair lists groups/levels in a chosen primary variable and ask to choose at least two groups/levels to be compared. Then, users need to choose a variable for pair/block IDs (e.g., subjects IDs for pre and post treatments or body sites). Then, users need to choose to analyze from phylum to genus (default) for 16S rRNA data^29,59^ or from phylum to species for shotgun metagenomic data^60^. Then, MiPair compares two groups or more than two groups (across groups or a baseline group to each of the other groups) in each taxon (Fig. 1) as follows.

#### Two-group comparison

1. For CLR: The parametric Paired t-test or the non-parametric Wilcoxon signed-rank test (default)^47^ can be employed to see if two groups have the same distribution for each taxon (${H}_{0}$) or if they have different distributions (${H}_{1}$). MiPair applies the BH procedures^56^ to each taxonomic rank to control for FDR. MiPair visualizes the results using box plots and dendrograms.
2. For Proportion, Arcsine-root or Count (rarefied): The parametric Paired t-test, the non-parametric Wilcoxon signed-rank test^47^, or the non-parametric linear decomposition model (LDM) (default)^61^ can be employed. MiPair applies the BH procedures^56^ to each taxonomic rank to control for FDR. MiPair visualizes the results using box plots and dendrograms.

#### More than two-group comparison (across groups)

1. For CLR: For the parametric inference, the repeated measures ANOVA F-test for global testing (to see if all groups have the same distribution for each taxon (${H}_{0}$) or if at least one group has a different distribution (${H}_{1}$)) with the Tukey’s HSD test^49^ for post-hoc comparisons (to test all possible pairs of groups individually) can be employed. For the non-parametric inference in complete block designs, the Friedman’s test^50^ for global testing with the Conover’s test^51^ for post-hoc comparisons (default) can be employed. For the non-parametric inference in incomplete block designs, the Durbin’s test for global testing with the Conover’s test^51^ for post-hoc comparisons (default) can be employed. MiPair applies the BH procedures^56^ to each taxonomic rank to control for FDR. MiPair visualizes the results using box plots and interactive volcano plots.
2. For Proportion, Arcsine-root or Count (rarefied): For the parametric inference, the repeated measures ANOVA F-test for global testing (to see if all groups have the same distribution for each taxon (${H}_{0}$) or if at least one group has a different distribution (${H}_{1}$)) with the Tukey’s HSD test^49^ for post-hoc comparisons (to test all possible pairs of groups individually) can be employed. For the non-parametric inference in complete block designs, the Friedman’s test^50^ for global testing with the Conover’s test^51^ for post-hoc comparisons can be employed. For the non-parametric inference in incomplete block designs, the Durbin’s test for global testing with the Conover’s test^51^ for post-hoc comparisons can be employed. For the non-parametric inference in either incomplete or complete block designs, LDM (default)^61^ can be employed for both global testing and pairwise comparisons. MiPair applies the BH procedures^56^ to each taxonomic rank to control for FDR. MiPair visualizes the results using box plots and interactive volcano plots.

#### More than two-group comparison (baseline to other groups)

For either CLR, Proportion, Arcsine-root or Count (rarefied), the likelihood ratio test (LRT) for global testing with the t-test for pairwise comparisons from a baseline group to each of the other groups based on LMM^52^ can be employed. MiPair applies the BH procedures^56^ to each taxonomic rank to control for FDR. MiPair visualizes the results using box plots and interactive volcano plots.

#### Example

**Figure 5:**
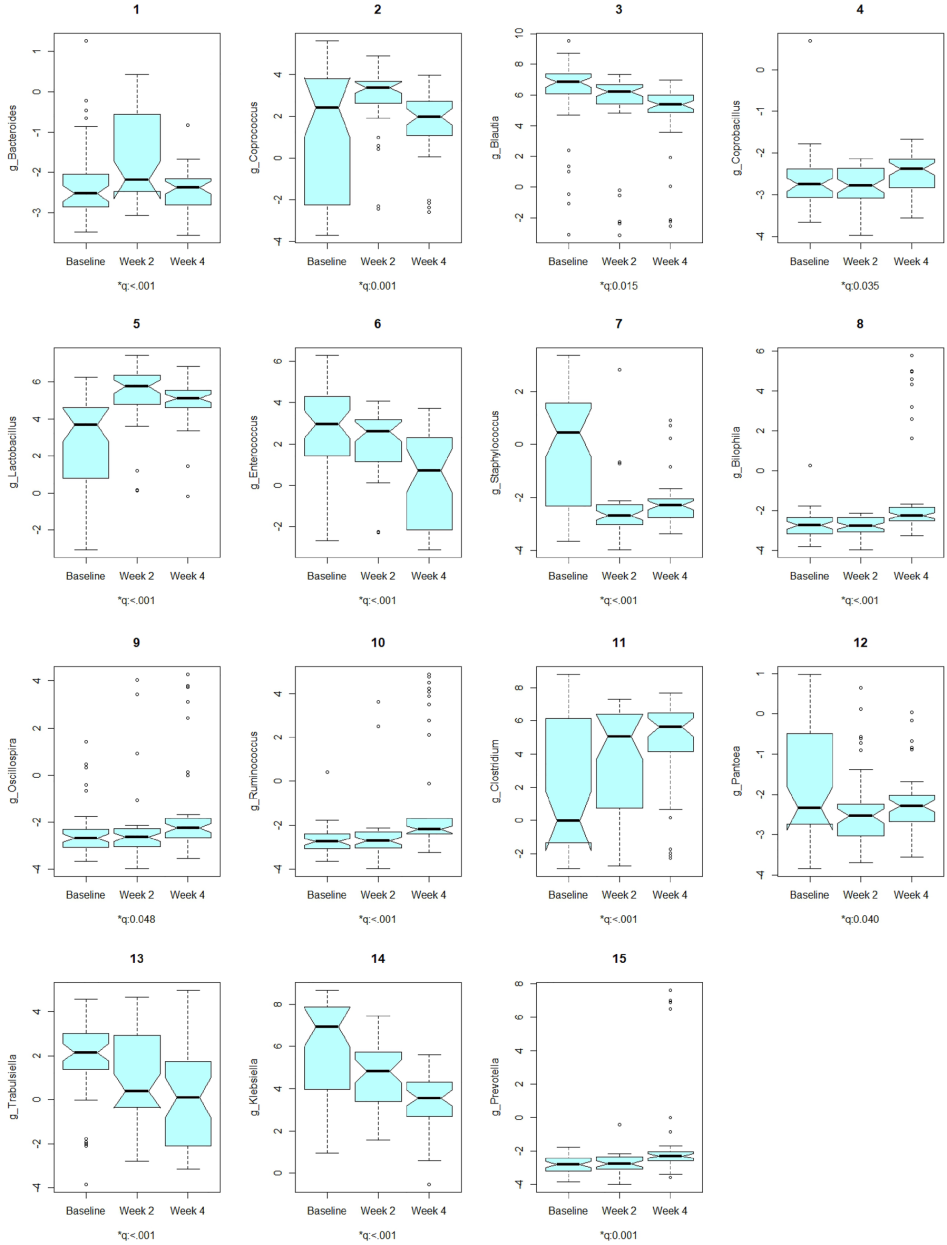
The 15 significant discoveries for comparitive analysis on genera (global test). The Q-values are the FDR adjusted P-values for global testing using LRT based on LMM to see if all groups have the same distribution in each genus (${H}_{0}$) or if at least one group has a different distribution in each genus index (${H}_{0}$).

**Figure 6:**
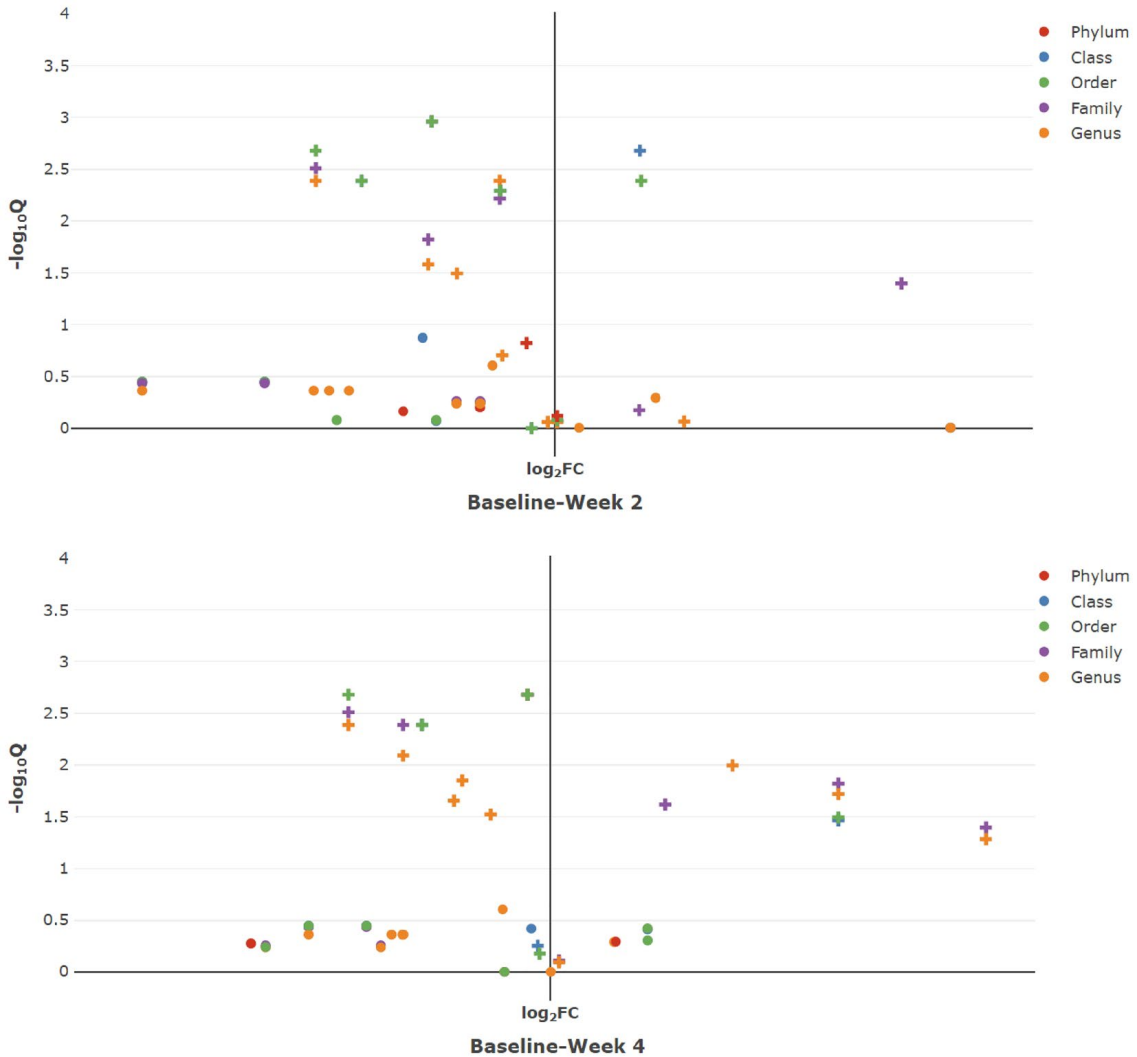
The volcano plot to overview the taxonomic differential abundances. Left: between the baseline group at the time of antibiotic administration and 2 weeks afterwards. Right: between the baseline group at the time of antibiotic administration and 4 weeks afterwards (right). x represents significantly differential taxa.

We chose CLR (default) as the data format to use and performed comparative analysis in each genus from the baseline group at the time of antibiotic administration to 2 weeks and 4 weeks afterwards^3^ using LMM for both global testing (Fig. 5) and pairwise comparisons (Table 3, Fig. 6). We found significant disparity in CLR transformed relative abundance for at least one group across the three groups for 15 genera at the significance level of 5% (Figs. 5, 6). Table 3 reports the results for those 15 genera in the context of pairwise comparisons between the baseline group and 2 weeks afterwards, and between the baseline group and 4 weeks afterwards, respectively.

**Table 3:** The results for comparitive analysis on genera (pairwise comparisons). *Ref represents the reference/baseline group, Com represents the comparison group, Est and SE represent the estimated regression coefficient and its standard error, t represents the t statistic value, and Adj. P-value represents the FDR adjusted P-value.

Genus	Ref	Com	Est	SE	t	Adj. P-value
g_Bacteroides	Baseline	Week 2	0.772	0.198	3.898	0.001
	Baseline	Week 4	− 0.201	0.200	− 1.004	0.389
g_Coprococcus	Baseline	Week 2	1.876	0.468	4.006	0.001
	Baseline	Week 4	0.597	0.474	1.258	0.290
g_Blautia	Baseline	Week 2	− 0.938	0.483	− 1.942	0.111
	Baseline	Week 4	− 1.519	0.490	− 3.099	0.012
g_Coprobacillus	Baseline	Week 2	− 0.127	0.100	− 1.270	0.285
	Baseline	Week 4	0.161	0.101	1.586	0.285
g_Lactobacillus	Baseline	Week 2	2.339	0.334	6.995	< 0.001
	Baseline	Week 4	1.892	0.339	5.579	< 0.001
g_Enterococcus	Baseline	Week 2	− 0.765	0.375	− 2.038	0.098
	Baseline	Week 4	− 2.705	0.380	− 7.109	< 0.001
g_Staphylococcus	Baseline	Week 2	− 2.504	0.314	− 7.973	< 0.001
	Baseline	Week 4	− 2.157	0.318	− 6.779	< 0.001
g_Bilophila	Baseline	Week 2	− 0.092	0.340	− 0.272	0.864
	Baseline	Week 4	1.552	0.344	4.514	< 0.001
g_Oscillospira	Baseline	Week 2	0.104	0.320	0.325	0.863
	Baseline	Week 4	0.793	0.325	2.442	0.067
g_Ruminococcus	Baseline	Week 2	0.195	0.351	0.555	0.751
	Baseline	Week 4	1.768	0.356	4.968	< 0.001
g_Clustridium	Baseline	Week 2	2.142	0.596	3.591	0.002
	Baseline	Week 4	3.057	0.605	5.050	< 0.001
g_Pantoea	Baseline	Week 2	− 0.568	0.221	− 2.573	0.062
	Baseline	Week 4	− 0.453	0.224	− 2.025	0.076
g_Trabulsiella	Baseline	Week 2	− 0.916	0.307	− 2.986	0.012
	Baseline	Week 4	− 1.663	0.312	− 5.332	< 0.001
g_Klebsiella	Baseline	Week 2	− 1.379	0.348	− 3.968	0.001
	Baseline	Week 4	− 2.654	0.353	− 7.529	< 0.001
g_Prevotella	Baseline	Week 2	0.048	0.362	0.131	0.896
	Baseline	Week 4	1.349	0.366	3.683	0.002

## Discussion

In this paper, we introduced an open-source web-based analytic tool, MiPair, for design-based comparative analysis with paired microbiome data. We described that MiPair can handle comprehensive microbiome data processing and analytic procedures using parametric or non-parametric tests for complete (in which every block contains all possible levels of treatments or body sites) or incomplete (in which not every block contains all possible levels of treatments or body sites) block designs to perform comparative analyses with respect to microbial ecology (alpha- and beta-diversity) and taxonomy (e.g., phylum, class, order, family, genus, species). We also described all the detailed widgets, methodologies and visualizations for the two-group comparison, more than two-group comparison (across groups) and more than two-group comparison (baseline to other groups), respectively.

We demonstrated the application of MiPair using an example clinical trial to see if the gut microbiome recovers from the time of a pulsed (macrolide) antibiotic administration to 2 weeks and 4 weeks afterwards, respectively^3^. However, the application of MiPair can be much broader. MiPair can be, in general, applied to any paired (block) designs, in which each pair/block contains different groups or levels of treatments. In the main text, we described subjects as example pairs or blocks for repeated measurements for different groups or levels of treatments or different body sites, yet twins or families can also be example pairs or blocks to control for genetic and/or environmental factors as in Refs.^9,12,62,63^. Besides, any groups of subjects that are matched in selected nuisance variables (e.g., age, sex) in an observational or quasi-experimental study can be pairs or blocks to control for such matched nuisance variables (e.g., age, sex) as in Refs.^64,65^. MiPair can substantially contribute to the rapidly growing human microbiome field as a useful and user-friendly data analytic tool for numerous potential applications.

## Materials and methods

### Web server, GitHub, URLs and pre-requisites

As in Ref.^28^, we constructed all the user interfaces and server functions of our app using R Shiny (https://shiny.rstudio.com), and distributed our app to web environments using ShinyProxy (https://www.shinyproxy.io) and Apache2 (https://httpd.apache.org). Our web server currently runs on Ubuntu 20.04 (https://ubuntu.com/) on the computing device with Intel Core i7-12700T (12-core) processor and 36 GB DDR4 memory allowing up to ten concurrent connections. We also set up a GitHub repository to allow users to run MiPair using their local computers in case that our web server is busy. We are the host that is responsible for maintaining our web server and GitHub repository stable. Users can report any issues that they have to us through the GitHub page (https://github.com/yj7599/mipairgit/issues).

#### URLs

MiPair is an open-source software, and can be reached through our web server (http://mipair.micloud.kr) or our GitHub repository (https://github.com/yj7599/mipairgit) locally on user’s computer.

#### Pre-requisites

MiPair depends on many other existing R packages, and thus it seems to require many pre-installations. However, users do not need to install them all individually because they are already installed on our web server. For the local device, they can also be installed and imported automatically using a simple command, library(shiny); shiny::runGitHub("mipairgit", "yj7599", ref = "main"), using the ‘shiny’ package on R Studio (https://www.rstudio.com). We have run unit tests using our web server with the specifications of Intel Core i7-12700T (12-core) processor and 36 GB DDR4 memory on Ubuntu 20.04 with R version 4.2.0, and also using two different local computers with the specifications of AMD Ryzen 7 5800U (8-core) processor and 8 GB DDR4 memory on Windows 11 Home (Version: 21H2, Build: 22000.1098) with R version 4.1.0 and the specifications of Apple M1 Ultra (20-core) processor and 64 GB memory on macOS Monterey 12.4 with R version 4.2.0, respectively. We have checked up each possible combination of the computing devices, datasets, and functionalities. For the datasets, we used the three example datasets^3^ and a huge synthetic dataset. The synthetic dataset was the one generated based on the Dirichlet-multinomial model^66^ using the estimated proportions and dispersion parameter of the gut microbiome data for the monozygotic twins in Ref.^9^. We generated the feature table for 6671 features and 3000 subjects, and created the metadata to have blocks with size three arbitrarily for the three-group comparison. Of course, the use of this synthetic dataset does not provide any biological or medical meanings at all. We used it just to check the running times for using such a huge dataset to provide some guideline on the upper limit of the data size that can be handled by MiPair. We organized the results from our unit tests in (Online resource, Supplementary Table 1). To summarize, we found no error for any procedure (Online resource, Supplementary Table 1). We also observed only small running times for any procedure for any of the three example datasets, yet we observed much greater running times for the huge synthetic dataset (Online resource, Supplementary Table 1). However, we would say that MiPair can still handle a huge dataset like the synthetic dataset with 6671 features and 3000 subjects in a manageable time. For the local device, we would also set up the minimum requirements as the one with 8-core processor and 8 GB memory on Windows or Macintosh with R (≥ 4.1.0). We monitor the capacity and functionality of our web server periodically. Users can also report any issues for using MiPair on our GitHub Issues page (https://github.com/yj7599/mipairgit/issues). We also plan to provide troubleshooting tips on our GitHub page (https://github.com/yj7599/mipairgit).

### Software packages

We wrote MiPair using R language, and MiPair is based on many R packages as follows.

#### Diversity calculation and data transformation

The alpha- and beta-diversity indices^35–46^ are calculated using the ‘phyloseq’, ‘picante’, ‘dist’, ‘ecodist’ and ‘GUniFrac’ packages. The CLR^57^ transformation and rarefaction^58^ are performed using the ‘compositions’ and ‘phyloseq’ packages.

#### Data analytic methods

The Paired t-test, Wilcoxon signed rank test^47^, and multivariate Hotelling’s t-squared test^48^ are performed using the ‘stats’ and ‘ICSNP’ packages. The ANOVA F-test, Friedman’s test^50^, Durbin test, Tukey’s HSD^49^ and Conover’s test^51^ are performed using the ‘stats’ and ‘PMCMRplus’ packages. The LMM^52^ is fitted using the ‘lme4’ package. The LDM^61^ is fitted using the ‘LDM’ package. PERMANOVA^53,54^ is performed using the ‘vegan’ package. The BH procedures^56^ are applied using the ‘stats’ package.

#### Visualizations

The box plots, histograms and forest plots are drawn using the ‘graphics’ and ‘forestplot’ packages. The PCoA plots^55^ are drawn using the ‘vegan’ package. The volcano plots are drawn using ‘plotly’ and ‘volcano3D’ packages.

## Supplementary Information

Supplementary Information.

## Data Availability

The raw sequence data for our example demonstration are publicly available in the database QIITA with the identifier 10508 (https://qiita.ucsd.edu/study/description/10508), and all the processed data components can be found on the app (see example datasets on Data Processing: Data Input). MiPair is an open-source software under the General Public License (GPL-1, GPL-2), which can be run on our web server (http://mipair.micloud.kr) or on user’s computer (https://github.com/yj7599/mipairgit).
