# Supplementary material for: Integrative web cloud computing and analytics using MiPair for design-based comparative analysis with paired microbiome data
Source: Sci Rep. 2022 Nov 28;12:20465. doi: 10.1038/s41598-022-25093-6 (PMC9705534; doi:10.1038/s41598-022-25093-6)
Supplement: Supplementary file 1 — Supplementary Information. [file 41598_2022_25093_MOESM1_ESM.pdf]

Supplementary Table 1. The results from our unit tests. The specifications for the devices we used are as follows: **Web Server:** Intel Core i7-12700T (12-core) processor and 36 GB DDR4 memory on Ubuntu 20.04 with R version 4.2.0; **Local 1:** AMD Ryzen 7 5800U (8-core) processor and 8 GB DDR4 memory on Windows 11 Home (Version: 21H2, Build: 22000.1098) with R version 4.1.0; **Local 2:** Apple M1 Ultra (20-core) processor and 64 GB memory on macOS Monterey 12.4 with R version 4.2.0. The datasets we used are as follows**: Example Data 1** contains 348 features for 87 subjects for a two-group comparison (a baseline group at the time of antibiotic administration and 2 weeks afterwards) (Zhang et al., 2018); **Example Data 2** contains 348 features for 120 subjects for a three-group comparison (a baseline group at the time of antibiotic administration and 2 weeks and 4 weeks afterwards) based on a complete block design (Zhang et al., 2018); **Example Data 3** contains 348 features for 128 subjects for a three-group comparison (a baseline group at the time of antibiotic administration and 2 weeks and 4 weeks afterwards) based on an incomplete block design (Zhang et al., 2018); **Synthetic Data** contains 6,671 features for 3,000 subjects for a three-group comparison based on a complete block design (Goodrich et al., 2014).

		**Web Server**	**Local 1**	**Local 2**
**Installation**		N/A (already installed)	OK (time: 23 sec)	OK (time: 22 sec)
**Implementation Using Example Data 1**				
Data Input	Unified Data	OK (time: 1 sec)	OK (time: 1 sec)	OK (time: 1 sec)
	Individual Data	OK (time: 1 sec)	OK (time: 1 sec)	OK (time: 1 sec)
Quality Control	Filtering/Visualization	OK (time: 1 sec)	OK (time: 1 sec)	OK (time: 1 sec)
Ecological Analysis	Diversity Calculation	OK (time: 2 sec)	OK (time: 2 sec)	OK (time: 2 sec)
	Paired t-test	OK (time: 1 sec)	OK (time: 1 sec)	OK (time: 1 sec)
	Wilcoxon signed-rank test	OK (time: 1 sec)	OK (time: 1 sec)	OK (time: 1 sec)
	Hotelling's t-squared test	OK (time: 1 sec)	OK (time: 1 sec)	OK (time: 1 sec)

	PERMANOVA	OK (time: 3 sec)	OK (time: 4 sec)	OK (time: 3 sec)
Taxonomic Analysis	Data Transformation	OK (time: 2 sec)	OK (time: 3 sec)	OK (time: 2 sec)
	Paired t-test	OK (time: 2 sec)	OK (time: 3 sec)	OK (time: 2 sec)
	Wilcoxon signed-rank test	OK (time: 2 sec)	OK (time: 3 sec)	OK (time: 2 sec)
	LDM	OK (time: 12 sec)	OK (time: 15 sec)	OK (time: 13 sec)
**Implementation Using Example Data 2**				
Data Input	Unified Data	OK (time: 2 sec)	OK (time: 2 sec)	OK (time: 2 sec)
	Individual Data	OK (time: 2 sec)	OK (time: 2 sec)	OK (time: 2 sec)
Quality Control	Filtering/Visualization	OK (time: 2 sec)	OK (time: 2 sec)	OK (time: 2 sec)
Ecological Analysis	Diversity Calculation	OK (time: 2 sec)	OK (time: 2 sec)	OK (time: 2 sec)
	ANOVA F-test/Tukey’s HSD	OK (time: 3 sec)	OK (time: 3 sec)	OK (time: 3 sec)
	Friedman’s test/Conover’s test	OK (time: 2 sec)	OK (time: 3 sec)	OK (time: 3 sec)
	LMM	OK (time: 2 sec)	OK (time: 3 sec)	OK (time: 2 sec)
	PERMANOVA	OK (time: 23 sec)	OK (time: 28 sec)	OK (time: 24 sec)
Taxonomic Analysis	Data Transformation	OK (time: 2 sec)	OK (time: 2 sec)	OK (time: 2 sec)
	ANOVA F-test/Tukey’s HSD	OK (time: 3 sec)	OK (time: 5 sec)	OK (time: 3 sec)
	Friedman’s test/Conover’s test	OK (time: 2 sec)	OK (time: 3 sec)	OK (time: 2 sec)
	LDM	OK (time: 37 sec)	OK (time: 39 sec)	OK (time: 38 sec)

	LMM	OK (time: 5 sec)	OK (time: 6 sec)	OK (time: 5 sec)
**Implementation using Example Data 3**				
Data Input	Unified Data	OK (time: 2 sec)	OK (time: 2 sec)	OK (time: 2 sec)
	Individual Data	OK (time: 2 sec)	OK (time: 2 sec)	OK (time: 2 sec)
Quality Control	Filtering/Visualization	OK (time: 2 sec)	OK (time: 2 sec)	OK (time: 2 sec)
Ecological Analysis	Diversity Calculation	OK (time: 2 sec)	OK (time: 2 sec)	OK (time: 2 sec)
	ANOVA F-test/Tukey's HSD	OK (time: 3 sec)	OK (time: 4 sec)	OK (time: 3 sec)
	Durbin's test/Conover's test	OK (time: 2 sec)	OK (time: 3 sec)	OK (time: 2 sec)
	LMM	OK (time: 2 sec)	OK (time: 2 sec)	OK (time: 2 sec)
	PERMANOVA	OK (time: 31 sec)	OK (time: 36 sec)	OK (time: 32 sec)
Taxonomic Analysis	Data Transformation	OK (time: 2 sec)	OK (time: 2 sec)	OK (time: 2 sec)
	ANOVA F-test/Tukey's HSD	OK (time: 5 sec)	OK (time: 6 sec)	OK (time: 5 sec)
	Durbin's test/Conover's test	OK (time: 2 sec)	OK (time: 2 sec)	OK (time: 2 sec)
	LDM	OK (time: 38 sec)	OK (time: 41 sec)	OK (time: 39 sec)
	LMM	OK (time: 5 sec)	OK (time: 8 sec)	OK (time: 6 sec)
**Implementation Using Synthetic Data**				
Data Input	Unified Data	OK (time: 7 sec)	OK (time: 8 sec)	OK (time: 7 sec)

	Individual Data	OK (time: 7 sec)	OK (time: 8 sec)	OK (time: 7 sec)
Quality Control	Filtering/Visualization	OK (time: 23 sec)	OK (time: 28 sec)	OK (time: 22 sec)
Ecological Analysis	Diversity Calculation	OK (time: 31 min)	OK (time: 32 min)	OK (time: 30 min)
	ANOVA F-test/Tukey’s HSD	OK (time: 25 min)	OK (time: 28 min)	OK (time: 26 min)
	Friedman’s test/Conover’s test	OK (time: 8 sec)	OK (time: 10 sec)	OK (time: 6 sec)
	LMM	OK (time: 7 sec)	OK (time: 8 sec)	OK (time: 7 sec)
	PERMANOVA	OK (time: 25 min)	OK (time: 24 min)	OK (time: 25 min)
Taxonomic Analysis	Data Transformation	OK (time: 6 min)	OK (time: 7 min)	OK (time: 6 min)
	ANOVA F-test/Tukey’s HSD	OK (time: 32 min)	OK (time: 33 min)	OK (time: 33 min)
	Friedman’s test/Conover’s test	OK (time: 22 min)	OK (time: 23 min)	OK (time: 22 min)
	LDM	OK (time: 48 min)	OK (time: 54 min)	OK (time: 49 min)
	LMM	OK (time: 8 min)	OK (time: 9 min)	OK (time: 9 min)
